# Tequila vinasses acidogenesis in a UASB reactor with *Clostridium* predominance

**DOI:** 10.1186/s40064-015-1193-2

**Published:** 2015-08-14

**Authors:** E N Marino-Marmolejo, L Corbalá-Robles, R C Cortez-Aguilar, S M Contreras-Ramos, R E Bolaños-Rosales, G Davila-Vazquez

**Affiliations:** Biotecnología Médica y Farmacéutica, Centro de Investigación y Asistencia en Tecnología y Diseño del Estado de Jalisco, Av. Normalistas 800, Col. Colinas de la Normal, C.P. 44270 Guadalajara, Jalisco Mexico; Tecnología Ambiental, Centro de Investigación y Asistencia en Tecnología y Diseño del Estado de Jalisco, Av. Normalistas 800, Col. Colinas de la Normal, C.P. 44270 Guadalajara, Jalisco Mexico

**Keywords:** Stillage, Volatile fatty acid, Biohydrogen, DGGE, Agave

## Abstract

Tequila vinasses represent an acidic, highly concentrated pollutant effluent generated during the distillation step of Tequila production. Although acidogenesis of Tequila vinasses has been reported for some reactor configurations, a characterization of the bacteria present during this metabolic process is lacking in the literature. Hydraulic retention times (HRT) between 36 and 6 h and organic loading rates (OLR) from 5 to 30 g COD L^−1^ d^−1^ were assessed in a UASB reactor fed with Tequila vinasses. Results showed that OLR excerted a stronger effect (*p* ≤ 0.0001) on parameters such as gas production rate, pH, and acidity than HRT. While it was clear that shorter HRT were related to higher volatile fatty acid production levels. Figures above 2 L_gas_ L_reactor_^−1^ d^−1^ (where “gas” could be a mixture of methane and hydrogen) were attained only with an OLR as high as 30 g COD L^−1^ d^−1^. Bacterial identification of a sludge sample at the end of the experiment revealed that acid-tolerant microorganisms that remained in the reactor were exclusively affiliated to the *Clostridium* genera, being the first report of organisms identification for Tequila vinasses acidogenesis. These findings are relevant to the field of biotechnology since acidogenesis of Tequila vinasses using identified and studied microorganism abilities (i.e. *Clostridium* strains) presents the opportunity of optimizing processes intended for different metabolites production (butanol, volatile fatty acids, hydrogen, solvents).

## Background

Total production of the well known mexican spirit called *Tequila* was around 242.4 million liters in 2014 (CRT 2015b). With seasonal variations in the past decades, Tequila production in the last ten years has nearly doubled (CNIT 2015). Thus, this economic activity is very important for a mexican region comprising 181 municipalities that retain the protected apellation of origin for this representative beverage (CRT 2015a). Being a spirit, Tequila production generates distillery wastewater (i.e. vinasse) in the order of 7–12 L of vinasse per liter of tequila produced and it is characterised for having a brownish color, high content of organic matter, low pH and high dissolved and suspended solids concentration (Méndez-Acosta et al. 2010; Buitrón et al. 2014b; López-López et al. 2010). Therefore, an estimate of Tequila vinasses production in 2014 was around 2,424 million liters and it is considered that only a small fraction of this received treatment before discharging it to water bodies and soil. Few tequila producers have invested in acquiring wastewater treatment plants and installed facilities for reducing the environmental impact of these effluents, being the anaerobic digestion (AD) process the most successful core technology for removing high levels of organic matter together with the production of bioenergy in the form of biogas (Buitrón et al. 2014b). However, it is acknowledged that the AD process is characterized by a delicate syntrophic interaction and dependence between *Bacteria* and *Archaea*, which can be upset by changes in some operational parameters and environmental conditions such as pH, substrate concentration, organic loading rate, toxic and/or inhibitory compounds present in complex effluents such as vinasses (Chen et al. 2008; Pant and Adholeya 2007; Silva et al. 2013; Mendez-Acosta et al. 2011). A common phenomenon that occurs in anaerobic digestors treating tequila vinasses is known as acidification of the system (Ilangovan et al. 2000). This undesirable phenomenon for anaerobic digestion operators has received little attention so far, but it could be considered as an opportunity for hydrogen production and volatile fatty acids (VFA) recovery rather than being an avoidable and unwanted process condition in terms of organic matter reduction with biogas production.

Previous works have described limited biodegradation of tequila vinasses (Retes-Pruneda et al. 2014) or hydrogen and VFA production in batch (Espinoza-Escalante et al. 2008), discontinuous (Buitrón and Carvajal 2010; Espinoza-Escalante et al. 2009; Buitrón et al. 2014a) and continuous (Buitrón et al. 2014b) processes. A batch process was selected by Espinoza-Escalante et al. (2008) to study the effect of pretreatments (alkalinization, thermal treatment and sonication) on tequila vinasse acidogenesis. Total VFA production was between 3,000 and 7,000 mg L^−1^ with an optimum total hydrogen production of 1,217 mL (from a 540 mL reactor working volume). On the other hand, Espinoza-Escalante et al. (2009) studied the effect of three operation parameters (hydraulic retention time (HRT), pH and temperature) in semi-continuous experiments to optimise the production of CH_4_ and H_2_. The authors found the best results for hydrogen production at pH = 5.5, HRT = 5 days and temperature = 55°C, with total VFA production in the order of 154 g L^−1^ and cumulative H_2_ and CH_4_ production of 5,600 and 1,230 mL, respectively. These authors also suggested that other parameters such as alkalinity and organic load should be studied in further research.

Anaerobic sequencing batch reactors (AnSBR) had been reported by some authors as suitable systems for hydrogen and VFA production from tequila vinasses. Buitrón and Carbajal (2010), studied the effects of temperature (25 and 35°C), HRT of 12 and 24 h and initial substrate concentration (0.5–5 g of chemical oxygen demand (COD) L^−1^) on hydrogen production using an AnSBR. Among the factors studied, the most significant was the HRT, with the maximum hydrogen production at 35°C and HRT of 12 h. Regarding total VFAs production, the authors found that higher concentrations were attained at HRT of 24 h (1396 mg VFA L^−1^) compared to 12 h (77 mg VFA L^−1^). Further research by the same group was performed to study hydrogen and methane production from vinasses in two separate reactors (acidogenic + methanogenic) (Buitrón et al. 2014a). For acidogenesis, the authors performed two sets of experiments using two reactors (with different working volumes), in the first one they tested a HRT of 18 h with 0.5, 1.0 and 5.0 mg soluble COD L^−1^. The second reactor was operated at HRT = 6 h with higher substrate concentrations: 2, 6, 8, 12 and 16 g soluble COD L^−1^. In both reactors, temperature and pH were controlled at 35°C and 5.5, respectively. The performance of the reactor operated with the lowest HRT and higher substrate concentration (highest OLR) resulted in a volumetric hydrogen production rate (VHPR) of 57.4 mL_H2_ L^−1^ h^−1^ and yield of 118 mL_H2_ g COD^−1^. Acetic, propionic and butyric acids were the main VFA detected at a concentration of 160 mg total VFA L^−1^. A fixed bed reactor was recently studied to assess hydrogen production in a continuous regime (Buitrón et al. 2014b). The authors performed the experiments with a HRT = 4 h and gradually increased the vinasse feed to the reactor reaching a organic load of 51 g COD L^−1^ d^−1^ with 100% vinasse. Best result for hydrogen production rate was 72 mL_H2_ L^−1^ h^−1^. Total VFA under continuous regime was around 1756 mg COD L^−1^ with acetic acid being the 15.8%, while butyric and i-butyric acids were 29.1 and 27.7%, respectively.

These previous reports had shown that it is feasible to obtain VFA and an energy carrier (biogas and/or hydrogen) from anaerobic digestion of tequila vinasses. However, reported results for VFA production in batch, semi-continuous and continuous regime, are in a wide interval going from 16 mg L^−1^ to 154 g L^−1^, therefore it is natural to expect that the performance of the systems is dependent on different operation parameters and regime (Davila-Vazquez et al. 2008; Bengtsson et al. 2008). Besides, to the best of our knowledge there is no information regarding the microorganisms that could be responsible for acidogenesis of a complex wastewater such as Tequila vinasses, moreover recent reports also suggested that this characterization could be process-dependent (Hernández-Mendoza et al. 2014).

Thus, the aim of this work was the assessment of hydraulic retention time (HRT) and organic loading rate (OLR) on the acidogenesis of Tequila vinasses during a continuous regime using a UASB reactor. The bacterial community adapted and therefore present in the reactor at the end of the experiment was analyzed using polymerase chain reaction–denaturing gradient gel electrophoresis (PCR–DGGE) and the volatile fatty acids produced were analyzed as well.

## Methods

### Tequila vinasses

A batch of 200 liters of tequila vinasse was obtained from a Tequila factory located in Guadalajara, Jalisco, Mexico. The batch was kept at 4°C prior it was utilized in the experiments. This factory produces “Tequila 100% Agave” which according to mexican regulation (NOM-006-SCFI-2012) means that all sugars present in the fermentation broth are derived from the Agave head (*piña*). A characterization of the tequila vinasse was performed according either to the *Standard Methods* (APHA et al. 1998) or HACH^®^ protocols (Loveland, Colorado, USA). Soluble COD was 40,786 mg L^−1^, pH = 3.81, Acidity 3,200 mg CaCO_3_ L^−1^, Conductivity 3.01 mS cm^−1^, Total phosphorous 1,204 mg L^−1^, Total nitrogen 174.6 mg L^−1^, Sulfates 103.8 mg L^−1^.

### Experimental procedure

A jacketed 1.2 L Upflow Anaerobic Sludge Blanket (UASB) glass reactor was used for performing the continuous experiments. The experimental scheme was intended to start with the longest HRT (36 h) and then gradually diminish it to 24, 12, and 6 h. Except for 36 h, at least in each HRT two different OLR were assessed. The reactor was maintained at 37 ± 1°C circulating water from a heated bath.

The biogas produced was conducted through a NaOH 2 M gas trap for CO_2_ elimination and after this step it was colected in an graduated acrylic glass cylinder used as a Mariotte bottle. Granular sludge from a full scale UASB reactor intended to Tequila vinasse methanisation was used as inoculum. A 400 mL granular sludge volume was used, with a volatile solids content of 4.13 g L^−1^. Anaerobic granular sludge used as inoculum seed received no previous treatment (e.g. heat shock, pH change).

A mineral nutrient solution was added to the feed vinasse solution to keep the concentration as follows: MgCl_2_·6H_2_O 10 mg L^−1^; MnSO_4_·6H_2_O 1.5 mg L^−1^; FeSO_4_·7H_2_O 2.5 mg L^−1^; CuSO_4_·5H_2_O 0.5 mg L^−1^; CoCl_2_ 0.3 mg L^−1^; Na_2_MoO_4_-2H_2_O 1.25 mg L^−1^; ZnCl_2_ 0.075 mg L^−1^; NH_4_Cl 3.0 g L^−1^; KH_2_PO_4_ 7.85 g L^−1^; K_2_HPO_4_ 7.37 g L^−1^. All chemicals were purchased from Sigma-Aldrich (St. Louis, Missouri, USA) as reagent grade.

Samples (10 mL) from the reactor effluent were withdrawn every 24 h for pH, acidity and alkalinity determination by titration methods (APHA et al. 1998). COD was measured three times a week with HACH^®^ reagents tests (Method 8000). Samples (3 mL) for VFA determination were taken three times a week and were preserved according to Park et al. (2005) and kept at 4°C until analysis by gas chromatography.

Volatile fatty acids (VFA) were analyzed by injecting a 0.5 μL sample into a gas chromatograph G1530 equipped with a flame ionization detector (Agilent, Wilmington, USA) and a capillary column DB-FAP (30 m × 0.25 mm i.d. × 0.25 μm film thickness; Agilent, Wilmington, USA). Helium was used as carrier gas at a flow rate of 1.0 mL min^−1^. Temperatures for the injector and flame ionization detector (FID) were 210 and 240°C, respectively. The VFA analyses were performed with a split ratio of 1:20 and a temperature program starting at 80°C for 1 min, increased gradually to 120°C (20°C min^−1^), and then it was warmed to a final temperature of 205°C (at 6 min^−1^).

Analysis of variance (ANOVA) were performed using Statgraphics ver. Centurion XVI (Statpoint Technologies).

### Microbial analysis by PCR-DGGE of partial 16S rRNA genes

#### DNA extraction

A 15 mL sludge sample was withdrawn from the bottom of the UASB reactor at the end of the experiment and was stored at −20°C until analysis. Reactor sample was slowly thawed and centrifuged (10,000 rpm, 10 min, 4°C) to obtain a 200–300 mg sludge pellet, the resulting pellet was washed three times by re-suspension with one milliliter of sterile phosphates–buffered saline buffer pH 7.5 and centrifugation of each preparation. DNA extraction was performed using the ZR Soil Microbe DNA MiniPrep™ (Zymo Research, California, USA) according to manufacturer instructions. A DNA integrity analysis was performed in 1% (w/v) agarose gels, stained with GelRed™ stain (Biotium, California, USA).

#### PCR amplification

Amplification of the hypervariable 3 region of the 16S rRNA gene from the purified nucleic acids preparations was carried out by PCR reactions and thermal programs where performed as previously described (Davila-Vazquez et al. 2011), the PCR primers used were the forward primer C356F (5′-CTACGGGAGGCAGCAG-3′) and the reverse primer 517R (5′-ATTACCGCGGCTGCTGG-3′). The primer C356F contains the GC clamp led to carry out the DGGE. The PCR product was loaded onto a 1.5% (w/v) agarose gel with 100 bp molecular weight marker and stained with GelGreen™ stain (Biotium, California, USA) to assess the size (236 bp), purity and concentration of DNA.

#### DGGE analysis

DGGE was performed with DCode™ Universal Mutation Detection System (Biorad, California, USA). The PCR products were loaded onto 10% polyacrylamide gels in 1 × TAE buffer (20 mM Tris, 10 mM glacial acetic acid, and 0.5 mM EDTA pH 8.0) with a denaturing gradient (urea-formamide) that ranged from 40 to 70%. Electrophoresis was carried out at 60°C and a constant voltage of 70 V was applied during 14 h. After electrophoresis the gel was stained using GelGreen™ for 30 min (Biotium, California, USA) before being visualized on a UV transilluminator (Biorad, California, USA). The dominant bands were excised from the gel, eluted in 10 mM Tris–HCl, 50 mM KCl, 1.5 mM MgCl, 0.1% Triton-x 100, pH 9.0, at 95°C for 20 min and centrifuged (10,000 rpm, 5 min). The DNA was reamplified by PCR with the conditions mentioned in the “PCR amplification” section. The PCR products from reamplification were purified and sent to sequencing at Macrogen Service Center (Maryland, USA). Sequence data were analyzed with BioEdit *v* 7.1 software (Ibis Bioscience, California, USA) and submitted to the non-redundant nucleotide data base at GenBank^®^ using the BLAST program (http://www.ncbi.nlm.nih.gov/blast/) and Ribosomal Database Project (http://rdp.cme.msu.edu/index.jsp) for bacterial identification.

## Results

### Reactor performance

**Fig. 1:**
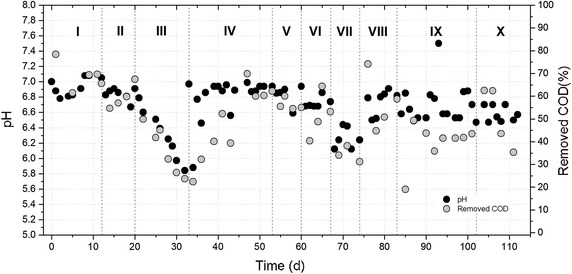
pH and organic matter removal during reactor operation. Experimental conditions were as follows, *I* (HRT = 36 h, OLR 5 g COD L^−1^ d^−1^), *II* (HRT = 24 h, OLR = 5 g COD L^−1^ d^−1^), *III* (HRT = 24 h, OLR = 15 g COD L^−1^ d^−1^), *IV* (HRT = 12 h, OLR = 5 g COD L^−1^ d^−1^), *V* (HRT = 12 h, OLR = 10 g COD L^−1^ d^−1^), *VI* (HRT = 12 h, OLR = 20 g COD L^−1^ d^−1^), *VII* (HRT = 12 h, OLR = 30 g COD L^−1^ d^−1^), *VIII* (HRT = 12 h, OLR = 5 g COD L^−1^ d^−1^), *IX* (HRT = 6 h, OLR = 5 g COD L^−1^ d^−1^), *X* (HRT = 6 h, OLR = 20 g COD L^−1^ d^−1^).

Performance of reactor in terms of pH and removal of chemical organic demand (COD) is shown in Fig. 1. Phases of operation (from I to X) together with operational conditions (HRT and OLR) are mentioned in the figure legend. Reactor was started to operate under a HRT of 36 h and an organic loading rate of 5 g COD L^−1^d^−1^. Results showed a COD removal percentage of 70% by the end of phase I (Fig. 1).

The combination of both HRT reduction from 36 to 24 h together with OLR increase from 5 to 15 g COD L^−1^d^−1^ led to a sharp decrease in both pH and removed COD (phase I, II and III, Fig. 1). Further reduction in HRT to 12 h (phase IV) and diminishing the OLR to 5 g COD L^−1^d^−1^ led to a system recovery in terms of removed COD from around 23% to 65–70% in a 2 weeks period. During this adaptation period (phase III to phase IV), the pH showed a faster response to the new operational parameters. Steady values were reached within 5 days (from pH 5.6 to 7.0). From phase IV to VII, HRT was kept at 12 h and OLR was augmented graduately from 5 to 30 g COD L^−1^d^−1^. During this period, both pH and removed COD showed a decrease from 7.0 to 6.1 in pH and from around 60 to 35% in COD removal. Due to an upset in the system, the reactor was started-up again in conditions mentioned for phase VIII (with the parameters figures of phase IV, Fig. 1). From this point, the HRT was reduced to 6 h and the OLR was set to 5 g COD L^−1^d^−1^. The pH value was then between 6.8 and 6.5 and figures for COD removal stabilized around 45%. During phase X, HRT was kept at 6 h and OLR increased to 20 g COD L^−1^d^−1^ which led to an average pH of 6.6 and after a recovery of 64%, COD removal showed a trend to diminish down to 35% at day 112.

The analysis of variance (ANOVA) to study the effect of HRT and OLR on volumetric gas production rate (VGPR) showed that OLR had a significant effect (*p* = 0.0001) whereas HRT (*p* = 0.1528) showed a weaker effect. As a consequence, and as it was expected, higher OLR resulted in higher figures of VGPR. As can be noticed in Table 1, figures above 2 L_gas_ L_reactor_^−1^ d^−1^ were attained only with an OLR as high as 30 g COD L^−1^ d^−1^. It is important to notice that gas identification analyses could not be performed, thus the term “gas” used here could be a mixture of CH_4_ and or H_2_.

**Table 1:** Effect of operational parameters on reactor performance

HRT (h)	VGPR^a^ (L_gas_ L_reactor_^−1^ d^−1^)	PH^a^	COD removal (%)^a^	Acidity^a^ (mg CaCO_3_ L^−1^)	Alkalinity^a^ (mg CaCO_3_ L^−1^)
6	1.09 ± 0.16 (25)	6.48 ± 0.06 (25)	36.04 ± 4.00 (12)	3,994.81 ± 183.29 (25)	282.54 ± 17.73 (25)
12	1.16 ± 0.12 (40)	6.57 ± 0.04 (40)	44.66 ± 2.72 (24)	4,120.14 ± 133.82 (40)	323.58 ± 12.94 (40)
24	1.62 ± 0.22 (16)	6.61 ± 0.07 (16)	54.26 ± 5.08 (10)	4,331.12 ± 242.05 (16)	337.73 ± 23.41 (16)
36	1.49 ± 0.23 (10)	6.72 ± 0.08 (9)	63.47 ± 5.64 (10)	3,615.65 ± 265.74 (9)	351.31 ± 25.71 (9)
OLR (g COD L^−1^d^−1^)					
5	0.91 ± 0.09 (55)	6.81 ± 0.03 (56)	55.05 ± 2.21 (30)	3,141.45 ± 103.10 (55)	293.92 ± 9.98 (55)
10	1.02 ± 0.28 (6)	6.85 ± 0.10 (5)	61.15 ± 6.19 (4)	3,135.29 ± 335.92 (5)	312.21 ± 32.49 (5)
15	0.90 ± 0.28 (9)	6.31 ± 0.09 (8)	31.86 ± 6.32 (6)	4,770.97 ± 311.63 (9)	331.12 ± 30.15 (9)
20	1.69 ± 0.18 (15)	6.71 ± 0.06 (15)	60.55 ± 4.47 (8)	3,592.49 ± 207.14 (15)	331.50 ± 20.04 (15)
30	2.18 ± 0.28 (6)	6.29 ± 0.10 (6)	39.43 ± 6.95 (3)	5,436.95 ± 311.53 (6)	350.21 ± 30.14 (6)

^a^Mean values are reported ± standard error, replicates for determination (n) are shown in parenthesis.

Similar results were obtained for statistical analysis regarding pH. For this parameter, OLR (*p* = 0.0001) exerted a stronger effect on pH than HRT (*p* = 0.0506). A clear trend in pH reduction is shown when diminishing HRT from 36 to 6 h (Table 1) achieving a pH = 6.48 with HRT = 6 h, although a lower figure (pH = 6.29) was observed with OLR = 30 g COD L^−1^ d^−1^. A pH-related parameter, acidity, was also affected by OLR (*p* = 0.0001) and in lesser extent by HRT (*p* = 0.1696).

The highest COD removal rates (around 70%) were obtained with an HRT of 36 h and OLR of 5 g COD L^−1^ d^−1^ (phase I) and an HRT of 12 h and OLR of 5 g COD L^−1^ d^−1^ (phase IV). The lowest COD removal rates (around 36%) were obtained with a HRT of 6 h (Table 1). This HRT promoted the production and accumulation of VFA in the system (4116.12 mg total VFA L^−1^; Table 2), reaching the highest VFA concentration in this experiment. The volumetric gas production obtained with a HRT of 6 h was 1.09 L_gas_ L_reactor_^−1^ d^−1^ (Table 1).

**Table 2:** Effect of operational parameters on volatile fatty acids production

HRT (h)	Acetic^a^ (mg L^−1^)	Propionic^a^ (mg L^−1^)	Butyric^a^ (mg L^−1^)	Valeric^a^ (mg L^−1^)	Isovaleric^a^ (mg L^−1^)
6	921.37 ± 18.62 (12)	783.03 ± 18.92 (12)	1,460.81 ± 19.09 (12)	375.78 ± 12.32 (12)	575.13 ± 21.46 (12)
12	866.94 ± 12.00 (29)	702.55 ± 12.19 (29)	1,131.95 ± 12.31 (29)	367.74 ± 7.94 (29)	536.30 ± 13.83 (29)
24	629.38 ± 23.95 (11)	453.61 ± 24.32 (11)	898.34 ± 24.56 (24)	265.73 ± 15.85 (11)	318.63 ± 27.60 (11)
36	593.57 ± 26.70 (5)	434.07 ± 27.12 (5)	722.87 ± 27.38 (5)	222.63 ± 17.67 (5)	211.95 ± 30.77 (5)
OLR (g COD L^−1^ d^−1^)					
5	700.11 ± 10.58 (31)	515.23 ± 10.75 (31)	970.73 ± 10.85 (31)	319.32 ± 7.00 (31)	421.89 ± 12.20 (31)
10	716.13 ± 29.65 (4)	560.87 ± 30.12 (4)	1,038.39 ± 30.40 (4)	313.63 ± 19.62 (4)	393.10 ± 34.17 (4)
15	873.36 ± 29.32 (7)	752.59 ± 29.78 (7)	1,123.09 ± 30.06 (7)	342.90 ± 19.40 (7)	588.47 ± 33.79 (7)
20	725.39 ± 20.51 (9)	587.44 ± 20.82 (9)	1,004.84 ± 21.02 (9)	276.83 ± 13.57 (9)	361.65 ± 23.63 (9)
30	749.08 ± 25.43 (6)	550.45 ± 25.83 (6)	1,130.41 ± 26.07 (6)	287.18 ± 16.83 (6)	287.40 ± 29.30 (6)

^a^Mean values are reported ± standard error, replicates for determination (n) are shown in parenthesis.

### Microbial analysis

**Fig. 2:**
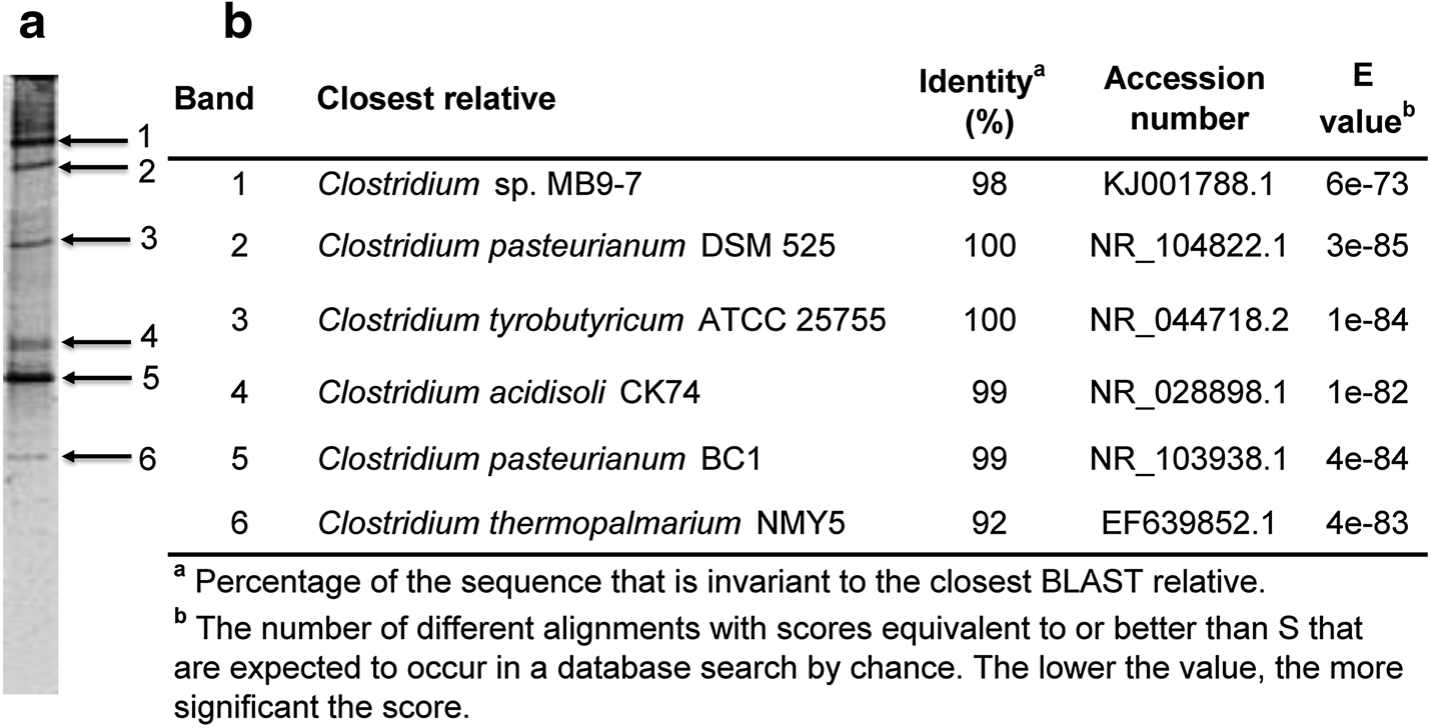
Bacterial identification of adapted microorganisms: **a** DGGE gel. **b** Identity of microorganisms present at the end of the experiment.

Results from bacterial analysis by PCR-DGGE of partial 16S rRNA genes are shown in Fig. 2. Six bands were successfully excised and DNA sequenced for identification of bacteria that adapted to process conditions and were present in the reactor at the end of the experiment. Bands numbered 1, 2, 3 and 5, resulted with the highest intensity in the DGGE gel (Fig. 2a), which is indicative of its relative abundance in this semi-quantitative analysis. Sequencing of the DNA retrieved from most intense bands resulted in the identification of *Clostridium* sp. MB9-7, *Clostridium pasteurianum* DSM 525, *Clostridium tyrobutyricum* ATCC 25755 and *Clostridium pasteurianum* BC1. Moreover, *Clostridium acidisoli* CK74 (Band 4) and *Clostridium thermopalmarium* NMY5 (Band 6), were also identified in the reactor biomass (Fig. 2).

## Discussion

As can be noticed from Fig. 1, there is a strong correlation in similar responses for pH and COD removal parameters during reactor operation (Fig. 1). This makes the pH an interesting parameter to predict the expected COD removal in future studies.

Regarding the operation parameters (HRT and OLR) in the reactor start-up, they are in accordance with the start-up values presented by Jauregui–Jauregui et al. (2014) for an anaerobic reactor fed with Tequila vinasses intended for methane production. Jauregui–Jauregui et al. (2014) obtained similar removal rates during the start-up of their reactor, with methane contents around 60–70% (v/v) under these conditions. When they reached an OLR of 5 g COD L^−1^ d^−1^, their HRT and COD removal were around 48 h and 70% respectively. In the present study, it was shown that this COD removal could be maintained at lower HRT values (12 h in phase IV).

In a recent study by Buitrón et al. (2014a), the authors assessed the hydrogen production performance in a SBR (sequencing batch reactor) using Tequila vinasses as substrate, with a HRT of 6 h. Under this condition they reached a peak hydrogen production of 57.4 mL_H2_ L_reactor_^−1^ h^−1^ (1.37 L_H2_ L_reactor_^−1^ d^−1^) with a COD removal of 18%. Further research by the same group (Buitrón et al. 2014b) improved their results using a fixed bed reactor under continuous regime obtaining 72 mL_H2_ L_reactor_^−1^ h^−1^ (1.72 L_H2_ L_reactor_^−1^ d^−1^) with a HRT of 4 h and a OLR of 51 gCOD L^−1^ d^−1^ achieving a carbon removal of 20%. As discussed by some authors, a short HRT is a selective pressure towards hydrogen-producing microorganisms selection, therefore it is suggested that volumetric hydrogen production rate obtained recently by other authors (Buitrón et al. 2014a, b) are comparable to our results under the HRT of 6 h. Despite the fact that gas identification could not be performed for our study, VFA levels, pH conditions achieved during bioreactor operation and the prevalence of *Clostridia* suggest that the main gas produced should be H_2_.

In a recent study (Wu et al. 2014), *Clostridium* sp. MB9-7 was genetically characterized and closely related to *C. tyrobutiricum*. Biochemical tests showed that this strain fermented glucose, xylose and mannose with butyrate, acetate, carbon dioxide and hydrogen as main products. On the other hand, *C. pasteurianum* BC1 was reported by Yarlagadda et al. (2012) as a fermentative organism which performed hydrogen, acetic and butyric acid production (acidogenesis) from glucose and could be directed to solventogenesis (ethanol and butanol production) with the aid of exogenous electron shuttles. Besides, it is noteworthy to mention that *C. pasteurianum* BC1 (ATCC 53464) was isolated from an acidic metal contaminated site, and has demonstrated efficient reduction of several redox-active metals in mineral salts medium. This strain was also found to be a rapid and efficient glucose fermenter compared to other clostridia strains, such as *Clostridium acetobutylicum* (ATCC 19403), *Clostridium sphenoides* (ATCC 19403), and *C. pasteurianum* (ATCC 7040) (Francis et al. 2008; Gao and Francis 2008). Moreover, *C. pasteurianum* DSM 525 which also was present in the system (Fig. 2) has been reported as an efficient butanol producer from thin stillage (Ahn et al. 2011). These authors demonstrated the feasibility of cost-effective butanol production by *C. pasteurianum* DSM 525 using thin stillage as a nutrient-containing medium.

On the other hand, *Clostridium acidisoli* CK74 was also identified in the reactor biomass (Fig. 2). This strain was studied by Kuhner et al. (2000) and characterized as nonacetogenic, N_2_-fixing, fermentative chemo-organotroph. The authors reported that this strain was isolated from acidic peat-bog soil, and had the ability to grow on substrates such as glucose, cellobiose, xylose, arabinose, maltose, mannose, salicin, mannitol, lactose, sucrose, glycerol, melezitose, raffinose and rhamnose. Growth of the strain on glucose yielded butyrate, lactate, acetate, formate, H_2_ and CO_2_ as end products. Showed growth at 5–37°C with an optimum between 25 and 30°C. Moreover, the doubling time on glucose was near 3.5 h (at pH = 4 and 30°C) (Kuhner et al. 2000).

Immobilized *C. tyrobutyricum* ATCC 25755 strain grown under continuous culture was studied by Mitchell et al. (2009). The effects of the hydraulic retention time (HRT = 8, 10, 12 or 16.7 h) and glucose concentration (30, 40 or 50 g L^−1^) on the production of hydrogen and butyrate were evaluated. Higher biogas and hydrogen production rates were generally seen when the HRT was lower. That study found the best conditions for the continuous production of hydrogen and butyric acid by *C. tyrobutyricum* to be with an HRT of 12 h and a glucose concentration of 50 g L^−1^, respectively. The previous reports for the *Clostridium* strains that were found in the reactor are important to consider new operation conditions for optimization in hydrogen production from Tequila vinasses. Our findings are relevant since acidogenesis of Tequila vinasses using identified and studied microorganism abilities (i.e. *Clostridium* strains) presents the opportunity of considering the process different than a “black box” concept and to optimize it depending on the microorganism and process conditions for different metabolites production (butanol, VFA, hydrogen, solvents).

The identification of *Clostridium* species only suggests that gas volumes reported here could be hydrogen-enriched streams which remains to be verified in future studies. It is noteworthy to mention that both inoculum selection and probably acidogenic conditions in the UASB reactor selected microorganisms that are capable to grow on acidic conditions (*C.pasteurianum* BC1, *C. acidisoli* CK74) and also with the ability to utilize a wide variety of substrates; both abilities crucial for being able to metabolise Tequila vinasses, a complex acidic wastewater.

## Conclusions

Volatile fatty acids (VFA) were produced from Tequila vinasses in a continuous regime with results similar to other reports using semi-continuous and continuous processes with the same class of substrate but different reactor configuration.

The most relevant from our report is that bacterial identification of the sludge sample at the end of the experiment revealed that acid-tolerant microorganisms that remained in the reactor were exclusively affiliated to the *Clostridium* genera, being this the first report of organisms identification responsible for Tequila vinasses acidogenesis. These findings are relevant to the field of biotechnology since acidogenesis of Tequila vinasses using identified and studied microorganism abilities (i.e. *Clostridium* strains) presents the opportunity of optimizing different processes intended for different metabolites production (butanol, VFA, hydrogen, solvents).

## References

[CR1] Ahn JH, Sang BI, Um Y (2011). Butanol production from thin stillage using *Clostridium pasteurianum*. Bioresour Technol.

[CR2] APHA, AWWA, WEF (1998) Standard methods for the examination of water and wastewater. 20th edn. American Public Health Association (APHA), American Water Works Association (AWWA), Water Environment Federation (WEF), Washington DC

[CR3] Bengtsson S, Hallquist J, Werker A, Welander T (2008). Acidogenic fermentation of industrial wastewaters: effects of chemostat retention time and pH on volatile fatty acids production. Biochem Eng J.

[CR4] Buitrón G, Carvajal C (2010). Biohydrogen production from Tequila vinasses in an anaerobic sequencing batch reactor: effect of initial substrate concentration, temperature and hydraulic retention time. Bioresour Technol.

[CR5] Buitrón G, Kumar G, Martinez-Arce A, Moreno G (2014). Hydrogen and methane production via a two-stage processes (H_2_-SBR + CH_4_-UASB) using tequila vinasses. Int J Hydrogen Energ.

[CR6] Buitrón G, Prato-Garcia D, Zhang A (2014). Biohydrogen production from tequila vinasses using a fixed bed reactor. Water Sci Technol.

[CR7] Chen Y, Cheng JJ, Creamer KS (2008). Inhibition of anaerobic digestion process: a review. Bioresour Technol.

[CR8] CNIT (2015) Información básica de la industria tequilera. Cámara Nacional de la Industria Tequilera (CNIT). http://www.tequileros.org/stuff/file_estadistica/1405441294.pdf. Accessed 20 Feb 2015

[CR9] CRT (2015a) Geography: the territory of the appellation of origin, or TDO. Consejo Regulador del Tequila (CRT). http://www.crt.org.mx/index.php?option=com_content&view=article&id=175&Itemid=185&lang=en. Accessed 20 Feb 2015

[CR10] CRT (2015b) Información Estadística. Consejo Regulador del Tequila (CRT). http://www.crt.org.mx/EstadisticasCRTweb/. Accessed 20 Feb 2015

[CR11] Davila-Vazquez G, Arriaga S, Alatriste-Mondragón F, de León-Rodríguez A, Rosales-Colunga LM, Razo-Flores E (2008). Fermentative biohydrogen production: trends and perspectives. Rev Environ Sci Biotechnol.

[CR12] Davila-Vazquez G, de León-Rodríguez A, Alatriste-Mondragón F, Razo-Flores E (2011). The buffer composition impacts the hydrogen production and the microbial community composition in non-axenic cultures. Biomass Bioenerg.

[CR13] Espinoza-Escalante F, Pelayo-Ortiz C, Gutiérrez-Pulido H, González-Álvarez V, Alcaraz-González V, Bories A (2008). Multiple response optimization analysis for pretreatments of Tequila’s stillages for VFAs and hydrogen production. Bioresour Technol.

[CR14] Espinoza-Escalante F, Pelayo-Ortíz C, Navarro-Corona J, González-García Y, Bories A, Gutiérrez-Pulido H (2009). Anaerobic digestion of the vinasses from the fermentation of *Agave tequilana* Weber to tequila: the effect of pH, temperature and hydraulic retention time on the production of hydrogen and methane. Biomass Bioenerg.

[CR15] Francis AJ, Dodge CJ, Gillow JB (2008). Reductive dissolution of Pu(IV) by *Clostridium* sp. under anaerobic conditions. Environ Sci Technol.

[CR16] Gao W, Francis AJ (2008). Reduction of uranium(VI) to uranium(IV) by clostridia. Appl Environ Microbiol.

[CR17] Hernández-Mendoza CE, Moreno-Andrade I, Buitrón G (2014). Comparison of hydrogen-producing bacterial communities adapted in continuous and discontinuous reactors. Int J Hydrogen Energ.

[CR18] Ilangovan K, Linerio J, Briones R, Noyola A, Olguin EJ, Sanchez G, Hernandez E (2000). Anaerobic treatment of tequila vinasse. Environmental biotechnology and cleaner bioprocesses.

[CR19] Jauregui-Jauregui JA, Mendez-Acosta HO, Gonzalez-Alvarez V, Snell-Castro R, Alcaraz-Gonzalez V, Godon JJ (2014). Anaerobic treatment of tequila vinasses under seasonal operating conditions: start-up, normal operation and restart-up after a long stop and starvation period. Bioresour Technol.

[CR20] Kuhner CH, Matthies C, Acker G, Schmittroth M, Gossner AS, Drake HL (2000). *Clostridium akagii* sp nov and *Clostridium acidisoli* sp nov.: acid-tolerant, N_2_-fixing clostridia isolated from acidic forest soil and litter. Int J Syst Evol Micr.

[CR21] López-López A, Davila-Vazquez G, León-Becerril E, Villegas-García E, Gallardo-Valdez J (2010). Tequila vinasses: generation and full scale treatment processes. Rev Environ Sci Biotechnol.

[CR22] Mendez-Acosta HO, Garcia-Sandoval JP, Gonzalez-Alvarez V, Alcaraz-Gonzalez V, Jauregui-Jauregui JA (2011). Regulation of the organic pollution level in anaerobic digesters by using off-line COD measurements. Bioresour Technol.

[CR23] Méndez-Acosta HG, Snell-Castro R, Alcaraz-González V, González-Álvarez V, Pelayo-Ortiz C (2010). Anaerobic treatment of Tequila vinasses in a CSTR-type digester. Biodegradation.

[CR24] Mitchell RJ, Kim JS, Jeon BS, Sang BI (2009). Continuous hydrogen and butyric acid fermentation by immobilized Clostridium tyrobutyricum ATCC 25755: effects of the glucose concentration and hydraulic retention time. Bioresour Technol.

[CR25] Pant D, Adholeya A (2007). Biological approaches for treatment of distillery wastewater: a review. Bioresour Technol.

[CR26] Park W, Hyun SH, Oh SE, Logan BE, Kim IS (2005). Removal of headspace CO_2_ increases biological hydrogen production. Environ Sci Technol.

[CR27] Retes-Pruneda JL, Davila-Vazquez G, Medina-Ramírez I, Chavez-Vela NA, Lozano-Alvarez JA, Alatriste-Mondragon F (2014). High removal of chemical and biochemical oxygen demand from tequila vinasses by using physicochemical and biological methods. Environ Technol.

[CR28] Silva FC, Serafim LS, Nadais H, Arroja L, Capela I (2013). Acidogenic fermentation towards valorisation of organic waste streams into volatile fatty acids. Chem Biochem Eng Q.

[CR29] Wu YF, Zheng H, Wu QL, Yang H, Liu SJ (2014). *Clostridium algifaecis* sp. nov, a novel anaerobic bacterial species from decomposing algal scum. Int J Syst Evol Microbiol.

[CR30] Yarlagadda VN, Gupta A, Dodge CJ, Francis AJ (2012). Effect of exogenous electron shuttles on growth and fermentative metabolism in *Clostridium* sp. BC1. Bioresour Technol.

